# Contributory factors to patient safety incidents in primary care: protocol for a systematic review

**DOI:** 10.1186/s13643-015-0052-0

**Published:** 2015-05-07

**Authors:** Sally Giles, Maria Panagioti, Andrea Hernan, Sudeh Cheraghi-Sohi, Rebecca Lawton

**Affiliations:** NIHR Greater Manchester Primary Care Patient Safety Translational Research Centre, University of Manchester, Suite 11, 7th floor, Williamson Building, Oxford Road, Manchester, M13 9PL UK; Centre for Primary Care, University of Manchester, Oxford Road, Manchester, M13 9PL UK; Greater Green Triangle University Department of Rural Health, Flinders University and Deakin University, PO Box 423, Warrnambool, Vic 3280 Australia; Institute of Psychological Sciences, Bradford Institute for Health Research (BIHR), University of Leeds, Leeds, LS2 9JT UK

**Keywords:** Patient safety, Primary care, Contributory factors

## Abstract

**Background:**

Organisations need to systematically identify contributory factors (or causes) which impact on patient safety in order to effectively learn from error. Investigations of error have tended to focus on taking a reactive approach to learning from error, mainly relying on incident-reporting systems. Existing frameworks which aim to identify latent causes of error rely almost exclusively on evidence from non-healthcare settings. In view of this, the Yorkshire Contributory Factors Framework (YCFF) was developed in the hospital setting. Eighty-five percent of healthcare contacts occur in primary care. As a result, this review will build on the work that produced the YCFF, by examining the empirical evidence that relates to the contributory factors of error within a primary care setting.

**Methods/design:**

Four electronic bibliographic databases will be searched: MEDLINE, Embase, PsycInfo and CINAHL. The database search will be supplemented by additional search methodologies including citation searching and snowballing strategies which include reviewing reference lists and reviewing relevant journal table of contents, that is, BMJ Quality and Safety. Our search strategy will include search combinations of three key blocks of terms. Studies will not be excluded based on design. Included studies will be empirical studies conducted in a primary care setting. They will include some description of the factors that contribute to patient safety. One reviewer (SG) will screen all the titles and abstracts, whilst a second reviewer will screen 50% of the abstracts. Two reviewers (SG and AH) will perform study selection, quality assessment and data extraction using standard forms. Disagreements will be resolved through discussion or third party adjudication. Data to be collected include study characteristics (year, objective, research method, setting, country), participant characteristics (number, age, gender, diagnoses), patient safety incident type and characteristics, practice characteristics and study outcomes.

**Discussion:**

The review will summarise the literature relating to contributory factors to patient safety incidents in primary care. The findings from this review will provide an evidence-based contributory factors framework for use in the primary care setting. It will increase understanding of factors that contribute to patient safety incidents and ultimately improve quality of health care.

## Background

**Figure 1:**
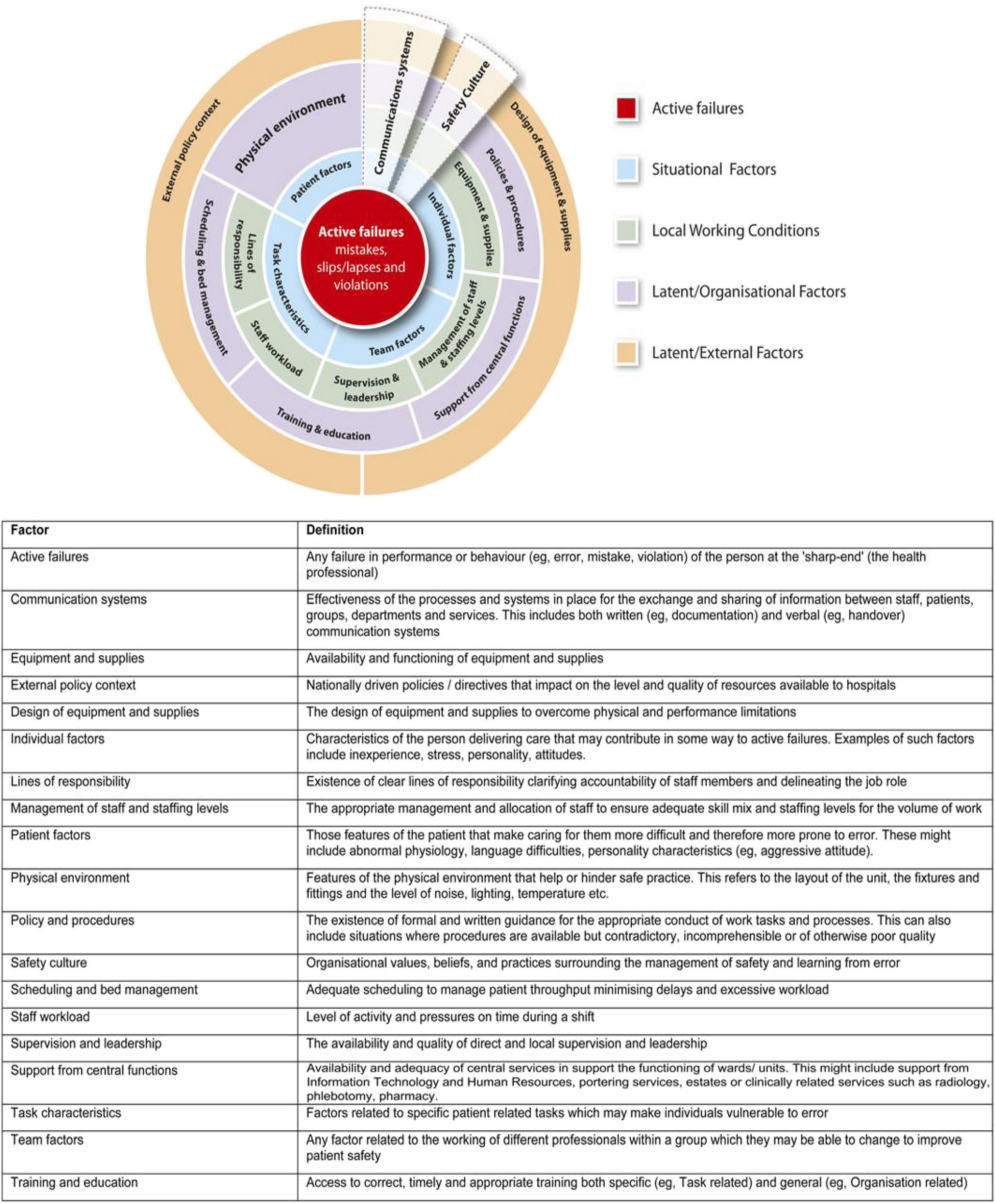
Yorkshire contributory factors framework.

Organisations need to systematically identify contributory factors (or causes) which impact on patient safety in order to effectively learn from error [1]. We define contributory factors as both proximal and latent causes of error, some examples of the contributory factors identified in hospital settings include communication, individual factors, physical environment and quality of treatment (see Figure 1). Investigations of error have tended to focus on taking a reactive approach to learning from error, mainly relying on incident-reporting systems. These systems have been criticised due to under-reporting [2] and a tendency to focus on the proximal causes of incidents [3,4]. Moreover, although there are a number of existing frameworks which aim to identify latent causes of error, for example, Eindhoven classification [5], WHO patient safety classification [6], the London Protocol [7], the Veterans Affairs Root Cause Analysis System [8], the Australian Incident Monitoring System (AIMS) [9], the LINNEAUS Patient Safety Classification for Primary Care [10] and a taxonomy of medical errors in family practice [11], these rely almost exclusively on evidence from non-healthcare settings [12,13], which are very different in structure to health care. There was clearly a need to develop an empirically based contributory factors framework specific to the healthcare context. This led to a recent systematic review that aimed to identify the factors that contribute to patient safety incidents within a hospital setting and to develop a contributory factors framework [14]. This framework is known as the Yorkshire Contributory Factors Framework (YCFF) and is the first of its kind (see Figure 1 for more details).

It is becoming increasingly recognised that patient safety research has tended to focus mainly on the secondary care setting, with comparatively little research within a primary care setting [15]. Given that 85% of all healthcare contacts occur in primary care [16], there is clearly a need to examine patient safety within a primary care setting. In addition, primary care is more diverse, has a broader scope and is varied in its structure and infrastructure which make it more vulnerable to error than a regulated hospital environment [11]. More recently, this has led to a greater focus on patient safety in a primary care setting, and the recognition that, although less is known about errors in primary care [17], errors in primary care are often very different from those found in a hospital setting [11]. Factors that contribute to error in a primary care setting tend to be more focused on the time between visits, poor care co-ordination amongst clinicians, the complexity of medication regimens and problems with access to services [17]. Given the obvious differences between the types of errors that occur in a hospital and primary care setting, there is a need to develop an evidenced-based contributory factors framework that is relevant to a primary care setting. This review will build on the work of Lawton *et al*. [14], by examining the empirical evidence that relates to the contributory factors of error within a primary care setting.

### Aims

This review aims to use a similar methodology that was used to develop the YCFF [14] but with a focus on primary care. We will use the YCFF as a guide to develop a framework in primary care. However, we anticipate that the contributory factors and the resulting framework of the current review will differ from the YCFF [14], given the differences in the types of error occurring between the hospital and primary care settings [11].

Consistent with the approach taken by Lawton *et al*., this systematic review has two objectives:

1. To identify factors contributing to patient safety incidents within a primary care setting.
2. To build a draft contributory factors framework for primary care. Further qualitative work will follow to test and further develop the framework.

## Methods/design

This review adheres to published guidelines for conducting and reporting systematic reviews [18]. The review is not registered with PROSPERO.

### Search strategy

Four electronic bibliographic databases will be searched: MEDLINE, Embase, PsycInfo and CINAHL. We will also identify eligible studies by checking the reference lists of those studies identified in the search that meet our inclusion criteria.

**Figure 2:**
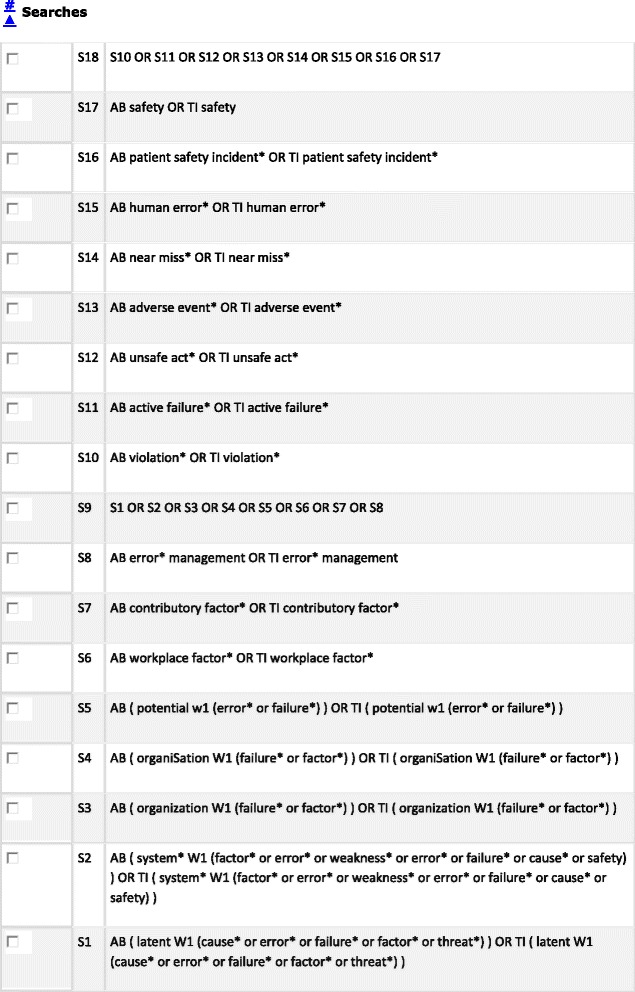
Search strategy.

Our search strategy will include search combinations of three key blocks of terms: *System/contributory Factors, Patient Safety* and *Primary Care*, similar to those used in the previous review [14], except the context is primary care. An example of our search strategy and the terms used (in MEDLINE) is listed in Figure 2.

### Eligibility criteria

Studies will be excluded if they fail to meet (a “NO” choice) any of the 3 criteria. Studies will be eligible for full-text screening if they fully (a “YES” choice to each criterion) or partly (one or more “UNSURE” choice) meet criteria A1, A2 and A3.

A. For any study type (including review articles and opinion pieces):
  1. Is it an empirical research?
    YES, NO, UNSURE
    Is it worth continuing?
  2. Does it make reference to contributory factors to patient safety incidents?
    YES, NO, UNSURE
    Is it worth continuing?
  3. The research has been conducted in primary care?
    YES, NO, UNSURE
    Is it worth continuing?

We will include:

- Types of studies: We will include empirical studies which provide data on factors that contribute to patient safety incidents in primary care. Study designs will not be restricted and will include both quantitative designs (that is, randomised controlled trials, quasi-experimental studies, cohort studies, cross-sectional studies) and qualitative studies including case studies. We will also include grey literature reports.
- Types of participants: patients in primary care. We will not exclude participants on the basis of age or diagnosis.
- Phenomena of interest: contributory factors of active failures or threats to patient safety. On the basis of the findings, an existing systematic review which examined contributory factors to patient safety incidents in secondary care settings, we anticipate that such contributory factors may include healthcare system/organisational factors (for example, communication failures between different professionals or patient-professional communication failures, staff workload, training/education, supervision/leadership, availability/use of equipment and supplies; policy issues; characteristics of the physical environment), health professional factors (inexperience, stress, personality attitudes) and patient factors (for example, language problems, personality characteristics, multimorbidity).
- Setting/context: We will focus on identifying studies conducted in primary care. We will also include studies conducted in the interface of primary and secondary care. We defined primary care as ‘the medical care involving first contact and on-going care to patients, regardless of the patient’s age, gender or presenting problem’ [19]. We will not restrict our search in specific geographical areas or date of publication.

We will exclude:

- Articles not published in English *(for pragmatic reasons such as translation difficulties)*
- Non-empirical studies *(primarily because we aim to build our conceptual framework based on empirical evidence rather than theoretical hypotheses and views that have not been empirically tested. Additionally, we do not expect to find any relevant systematic reviews of empirical studies given the lack of systematic evidence in this research area)*
- Studies that report only patient safety incidents without providing information on factors that may account for these incidents
- Studies relating to home care *(considered to be contextually different from general primary care settings).*

### Management of search outcomes and study eligibility screening

The results of the searches of each database will be exported to an Endnote reference management database (version X4) and merged to identify and delete duplicates.

We will use the taxonomy proposed by intra-EuropeanLINNEAUS Euro-PC collaboration for abstract and full-text screening. According to this taxonomy, patient safety incidents fall into three categories, access of health care (that is, incident related to availability, accessibility, accommodation, affordability and acceptability of health care), clinical task (that is, incident related to history/examination/problem identification, diagnosis, treatment, delivery, rehabilitation, prevention) and organisational task (that is, incident related to administration, supervision/management, maintenance, payment).

**Figure 3:**
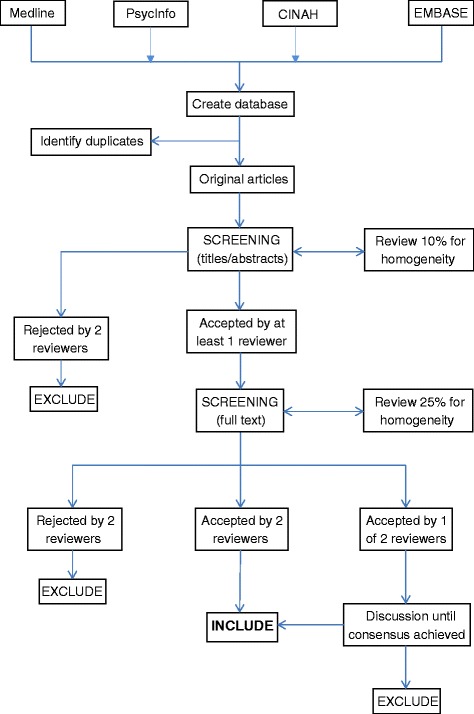
Screening process.

Using PRISMA guidelines [18], screening will be completed in two stages (see Figure 3). Initially, the titles and abstracts of the identified studies will be screened for eligibility (see ‘Eligibility criteria’ section). A proportion of titles and abstracts (50%) will be screened by two researchers independently to assess reliability using the kappa statistic. Assuming reliability is confirmed, screening of the remaining titles and abstracts will be completed by one reviewer.

### Eligibility criteria

Next, the full texts of studies initially assessed as ‘relevant’ for the review will be retrieved and checked against our inclusion/exclusion criteria. Full-text screening will be completed by two researchers independently, with disagreements resolved by discussion.

### Methodological quality of the studies

Quantitative studies: We expect the main body of the research included in this review to be observational studies (cross-sectional or prospective). Thus, for the main bulk of anticipated studies (observational studies), we decided to assess the methodological quality using criteria adapted from guidance on the assessment of observational studies [20]. The quality review will include assessment of the design, conduct and analysis of each study and will be used as a framework for the narrative synthesis of the results. Three key criteria will be used to conduct the quality review, and each study will be awarded one point for each criterion met These key criteria are:

1. A response rate of 70% or greater at baseline
2. Adequate control for confounding factors in analysis
3. A follow-up rate of greater than 70% (in prospective studies).

These criteria allow rapid evaluation and have been previously used by members of our research group to assess the methodological quality of observational studies [21]. The criteria will not be used to exclude papers prior to the synthesis; rather, it will be used to provide a context for the interpretation of the findings. The methodological appraisal of any randomised controlled trials (RCTs) identified in this review will be assessed using the Cochrane risk of bias assessment tool [20]. However, if a range of different types of experimental studies are identified including RCTs, non-randomised trials, controlled before after studies and time series studies, the methodological quality of those studies will be assessed using nine standardised criteria developed by the Effective Practice and Organisation of Care (EPOC) [17].

Qualitative studies: The methodological appraisal of qualitative studies indentified in this review will be assessed using the Critical Appraisals Skills Checklist (CASP) for qualitative studies [22].

Each paper will be independently appraised by two reviewers, and discrepancies will be resolved by discussion.

### Data extraction

A data extraction sheet will be devised that will include the following:

- Study characteristics - year, objective, research method, setting, country.
- Participant characteristics - number, age, gender, diagnoses.
- Patient safety incident type and characteristics
- Practice characteristics
- Main outcomes
  I. Contributory factors to patient safety in primary care
- Results of the study quality appraisal.

Data extraction will be completed by two researchers. Disagreement will be resolved by discussion until consensus is reached.

### Data synthesis

The outcomes of the systematic review will be organized and presented descriptively. The heterogeneity of the research designs and outcomes of this review are unlikely to allow the use of formal meta-analytic procedures.

On the basis of a previous paradigm in hospital settings [14], a contributory factor framework will be developed. Studies will be categorised by types/domains of contributory factors to patient safety (that is, communication failures, equipment and supply, active failures and so on) in primary care. Coding contributory factors into different domains will be conducted by two authors independently. In addition, expert advice (RL) will be sought about the contributory factor coding process. Depending upon the consistency in reporting methods and the number of studies retrieved, we aim to further group studies according to study design and methodological quality. This synthesis will be performed by the lead author (SG) and then reviewed independently by co-authors. Disagreements will be resolved by consensus.

## Discussion

The review aims to build on the work of another systematic review [14], conducted in the hospital setting by two of the authors on this protocol (Giles and Lawton). It will summarise the literature relating to contributory factors to patient safety incidents in primary care. The findings from this review will provide an evidence-based contributory factors framework for use in the primary care setting. It will increase understanding of factors that contribute to patient safety incidents and ultimately improve quality of health care.

## Abbreviations

AIMS: Australian Incident Monitoring System
WHO: World Health Organization
YCFF: Yorkshire Contributory Factors Framework
